# Annotations

**Published:** 1887-07-23

**Authors:** 


### Annotations.

Dishonourable Consultants.

There appears to be no doubt that the charge made against some consultants of retaining patients sent to them by family doctors is quite true. The following case occurred recently. A young man was sent by his ordinary practitioner to a certain consultant of standing, not from any doubt about the nature of the case or the treatment, but merely because the patient had been a long time ill, and appeared likely to continue so. No consultation had been asked for either by patient or friends, but the doctor himself, to satisfy his own conscience that the best which modern medicine could do for his patient was being done, suggested one. The consultant agreed entirely with the ordinary attendant, both as to diagnosis and treatment; and that being so, it was surely his duty to return the patient to the doctor who had sent him. Instead of doing so, he asked the young man to come up to him again from the country at a date named, without further reference to the ordinary attendant. The patient, who had clearly understood both from the remarks of the consultant and the prescription given, that there was an entire agreement in every particular between his family attendant and the greater light, returned to his first love, and told what had been said to him, adding that as there appeared to be nothing to gain by the change, he did not see why he should pay two guineas for each consultation instead of the modest five shillings charged by the family attendant. This little story bears its own moral.

Untrained Bathers.

John Ploughman, in the summer, may bathe in the sea when he likes, where he likes, and as often as he likes; Miss Finnicky, who lives in lodgings in town, has a cup of tea before rising in the morning, and dare not face the dangers of the streets without her smelling-bottle, should never bathe at all without first taking her doctor's advice. To the former a bold plunge into twenty feet of water is as invigorating as a brisk scent to a jaded hound: for the latter, the only safe course is to take a gentle and gingerly dip, holding on for safety by the wheel of the bathing-machine. Sea-bathing is a very powerful tonic for the strong, and a dangerously exhausting exercise for the weak. The undergraduate may, as a rule, enjoy it to his heart's content; the curate with a family, and the hard-worked barrister should take it with caution, and in strictly shallow water. Physical strength can only be maintained by physical exertions, and for all such exertions the average town man is generally out of training. His muscles are flabby and his wind is bad; and as a consequence, no sooner has he taken a dozen strokes in the water than both wind and muscle give out; and then, if he cannot float, he is in rather a bad way. In July and August nothing is better even for weakly people than sea-bathing, but it should always be taken with the utmost discretion. Begin with a gentle dip, and keep well within your depth. A little more depth next day, and a few strokes may be indulged in. If the weather be hot, a brief dip twice a day will do no harm. At the end of a week a short swim should not be difficult; and if progress be gradual, three weeks should be sufficient to turn you into an average athlete. For young ladies the most necessary piece of advice is—do not stay too long in the water. As a rule, from fifteen to thirty minutes should be the utmost limit. Much less is usually much better.

"Sir Oracle."

"Give a dog a bad name and hang him:" give him a good one, and he will often hang himself. Many a man has the pluck and perseverance to fight his way to a recognised position, but when he has reached it he has not the judgment, honesty, and weight of character which will enable him to retain it. Being convinced that because he has reached such an altitude, therefore he must be a great person, he begins to lay down the law in an infallible kind of way, instead of remaining as he was in the days before fame was reached,—a student one part of the day, and a teacher the other. The consciousness of infallibility is fatal to progress and to enduring worth. The man who is lifted up to a feeling of very high personal merit and extravagant personal claims by his success in life is essentially a small person, and cannot occupy a lasting niche in the temple of fame. It is with character and work as the Scripture says it is with religion,—"He that endureth to the end; the same shall be saved." The truly great man is not he who proclaims on the house-top that he is an oracle and a power, but he who, with the full consciousness of the infirmities which belong to him as to every other human being, strives to the last day of his life to do every duty thoroughly and perfectly because it is a duty, and takes such temporary and permanent rewards as the doing of such duty naturally brings. Oracles and self-trumpeters belong to the class of persons "to be suspected."

Lemon Juice.

As soon as the summer comes round everybody is asking, "What shall we drink?" It is a pity the answer to the question is so difficult. "Water," no doubt the abstainer would say, "is the natural quencher of the thirst." Agreed! but in consequence of the uncertainty as to the quality of water, and the state of many civilised stomachs, plain, uncooked, and unadulterated water is not always admissible. Among the pleasantest and most wholesome of fluids may be named lemon and lime juice. They do not suit everyone, neither do they possess those extraordinary virtues which some imaginative or interested persons are constantly claiming for them. But they are decidedly pleasant and undoubtedly conducive to health. A marked feature in their favour is that they can be manipulated in so many and such various ways. They may be taken sweetened or unsweetened, with water, claret, sherry, or whiskey, and, indeed, in an almost infinite variety of forms.

